# Editorial: The role of oxidative stress in metabolic and inflammatory diseases

**DOI:** 10.3389/fendo.2024.1374584

**Published:** 2024-02-08

**Authors:** Aleksandra Klisic, Rasheed Ahmad, Dania Haddad, Francesca Bonomini, Sardar Sindhu

**Affiliations:** ^1^ Department for Biochemistry, University of Montenegro-Faculty of Medicine, Podgorica, Montenegro; ^2^ Center for Laboratory Diagnostics, Primary Health Care Center, Podgorica, Montenegro; ^3^ Department of Immunology and Microbiology, Dasman Diabetes Institute, Dasman, Kuwait; ^4^ Genetics and Bioinformatics Department, Dasman Diabetes Institute, Dasman, Kuwait; ^5^ Anatomy and Physiopathology Division, Department of Clinical and Experimental Sciences, University of Brescia, Brescia, Italy; ^6^ Animal and Imaging Core Facilities, Dasman Diabetes Institute, Dasman, Kuwait

**Keywords:** oxidative stress, ROS, reactive oxygen species, metabolic diseases, inflammatory diseases

The imbalance in the redox homeostasis, caused by either an overexpression of Reactive Oxygen Species (ROS) or a decrease in antioxidant defense, sets the stage for deleterious chemical changes to occur in biomolecules such as lipids, nucleic acids (DNA/RNA), and proteins. ROS refer to highly reactive, oxygen-containing molecular species that include superoxide anion (O_2_^•−^), hydrogen peroxide (H_2_O_2_), and hydroxyl radical (HO^•^). ROS are primarily generated from molecular oxygen (O_2_) during the four successive steps of one-electron reduction in the mitochondrial respiratory chain. The free radicals (O_2_^•−^/HO^•^) can further react with organic substrates to form intermediate species or secondary ROS, such as peroxyl radical (RO_2_^•^) and hydroperoxide (ROOH). H_2_O_2_, which is relatively less toxic and more stable, can transform into highly toxic products as it interacts with Fe^++^ (Fenton reaction) or in the presence of O_2_^•−^ (Haber-Weiss reaction). Notably, about 90% of the endogenous ROS are generated during the process of oxidative phosphorylation in the mitochondria. These are cellular powerhouses responsible for producing energy in the form of ATP molecules, which fuel biochemical reactions and support biological functions. In addition to ATP synthesis and ROS production, mitochondria also play a role in regulating intracellular Ca^2+^, apoptosis, and the activation of caspase family proteases. ROS accumulation, whether due to overproduction or as a sequel to defective antioxidant defense, may lead to irreversible damage to the mitochondria, as often observed in metabolic and inflammatory diseases.

The aim of this Research Topic was to advance our current understanding about how the oxidative stress acts as a pathophysiological factor in meta-inflammatory diseases by modulating the critical pathways and signals that regulate the genetic and epigenetic landscape reprogramming in such disorders. Given that recent years have witnessed a substantial progress in developing newer, more effective therapeutic strategies, pharmacologic interventions, and mitochondria-targeted approaches that counter oxidative stress and improve mitochondrial function, the purpose was to highlight the recent advances and attempt to fill knowledge gaps for a clearer understanding of the causal or exacerbating role of free radical stress in meta-inflammatory disorders.

The current Research Topic provides an opportunity to present a handful of high-quality publications that highlight the potential role of oxidative stress in metabolic and inflammatory disease pathogenesis. This Research Topic published four highly important manuscripts, comprising three reviews and one original research article, as summarized in the following.

The Nuclear Factor Erythroid 2 (NFE2)-Related Factor 2 (NRF2) is a member of the Cap’n’Collar (CNC) subfamily of basic region leucine zipper transcription factors, and it plays a role as a master regulator of antioxidant defense by resisting oxidant stress, a function that is evolutionarily conserved across vertebrates (1). NRF2 induction requires a common DNA sequence known as the Antioxidant Response Element (ARE), which resembles the NFE2 binding motif. The identified NRF2 target genes that control redox homeostasis are found to contain the ARE motif. Under basal conditions, NRF2 expression is regulated by the “de-depression mechanism” and is suppressed by Kelch-like Erythroid Cell-Derived Protein with CNC Homology (ECH)-Associated Protein 1 (KEAP1)-dependent ubiquitination and proteasomal degradation. Conversely, NRF2 expression is induced by stress involving oxidants and electrophiles. In their elegant review paper titled “*The role of NRF2 in periodontal disease by regulating lipid peroxidation, inflammation and apoptosis*”, Ma et al. sift through the growing body of evidence, supporting that the anti-apoptotic, anti-oxidative, and anti-inflammatory attributes of NRF2 make it an attractive therapeutic target for periodontal disease. The authors review and update on the recent studies that decipher the link between oxidant stress and the development of periodontitis, as well as discuss the scope of treatment strategies that target the KEAP1-NRF2-ARE axis, e.g., using electrophilic NRF2 activators such as curcumin, sulforaphane, quercetin, and resveratrol. These agents exert antioxidant and anti-inflammatory effects. The authors allude to a growing need to move past the “proof-of-concept” level and venture into studies that further address the pharmacodynamic, clinical safety and efficacy evaluation of these candidates for non-communicable diseases marked by oxidant stress and inflammation. In this review, the authors also discuss ingenious drug delivery strategies such as solid dispersion, nano-formulation, and self-microemulsifying systems that could be used to improve drug bioavailability and pharmacokinetics. Based on recent evidence, the authors conclude that maintaining redox homeostasis by potentiating NRF2 signaling, coupled with early diagnosis of periodontal disease, could warrant a successful outcome of treatment and prevention strategies.

Notably, the prevalence of cerebrovascular diseases is persistently increasing in younger age groups the world over, requiring urgent testing of new and more effective approaches. As one of the potential drivers of cellular aging or senescence (a hallmark and intrinsic feature of all living beings), oxidative stress emerges as a critical player in the pathogenesis of neurodegenerative diseases (2). In their interesting review paper entitled: “*Oxidative stress in cerebrovascular disease and associated diseases*”, Kumar et al. present the current advancements and review the scientific evidence for a key role of oxidative stress in the progression of cerebrovascular diseases, especially in the pathogenesis of stroke. Additionally, the authors also discuss the contributory roles of other oxidative stress-associated factors such as hypertension, diabetes, cardiometabolic, and genetic factors that could impact stroke pathogenesis. Finally, the authors suggest that considering oxidative stress as a common root cause of pleiotropic effects, novel therapeutic and prevention strategies for stroke and related pathologies need to aim at re-establishing the antioxidant defense and restoring cellular homeostasis.

Building on the current understanding, infertility affects nearly 10-15% of couples worldwide, and among all infertility cases, 20-70% relate to male factors (3). Increasing evidence suggests that oxidative stress is a potential risk factor for male infertility, in addition to other factors such as obesity, diabetes, hypo- and hyperthyroidism. Melatonin (N-acetyl-5-methoxytryptamine) is an indoleamine produced in many organs, including the pineal gland. Studies over the past decade show that melatonin and its metabolites possess the ability to scavenge both oxygen and nitrogen-based reactive species (ROS/RNS) and block the transcriptional factors that drive the expression of proinflammatory cytokines and chemokines. Besides, it also stimulates the expression of other anti-oxidant enzymes, which, overall, makes it a potent broad-spectrum free radical scavenger that can regulate cellular homeostasis. Nonetheless, a critical overview hinged on emerging evidence has long been awaited supporting its therapeutic benefit for male infertility. In this elegant review paper entitled “*Protective effects of melatonin against oxidative stress induced by metabolic disorders in the male reproductive system: a systematic review and meta-analysis of rodent models*”, Ebrahimi et al. have systematically looked through the online databases, including PubMed, Scopus, and Web of Science, for studies evaluating the effects of melatonin therapy on metabolic disorders-induced infertility in male rats and mice. Among the 24 studies included in this review, metabolic disorders such as obesity, diabetes, hyperlipidemia, food deprivation, hypo- and hyperthyroidism were experimentally induced in rodents by using approaches like high-fat/high-fructose diets, alloxan, leptin, streptozotocin, carbimazole, and levothyroxine. The 31 outcomes chosen for meta-analyses included the histopathological characteristics of the testicular tissue, weight-related measurements, reproductive hormonal profiles, oxidative stress markers, and exploratory (gluco-lipid) parameters. Overall, pooled effects for the histopathological characteristics and some oxidative stress markers were found to be statistically significant, providing evidence that melatonin could limit damage to the gonadal tissue and improve sperm counts, motility, and morphology in metabolic disease-associated male infertility. This meta-analysis underscores the need for further clinical studies and randomized controlled trials, aiming at the safety and efficacy of melatonin for male infertility in individuals with metabolic disorders.

Obesity is a metabolic disorder marked by chronic low-grade inflammation, loss of glycemic control, and metabolic dysregulation. Besides inflammation, oxidative stress is another top player that is involved in metabolic dysregulation, as ROS are contributed mainly by increased pro-oxidant activities including oxidative phosphorylation, O_2_^•−^generation, and protein kinase C activation. Emerging evidence points to the adipotropic effects of heavy metals and metalloids and their potential role in pathophysiological changes in obesity, diabetes, or metabolic syndrome (4). Admittedly, studies that decipher the potential link between metals or metalloids, inflammation, and oxidative stress in obesity are direly needed. To this end, in the study entitled: “*Vanadium, cobalt, zinc, and rubidium are associated with markers of inflammation and oxidative stress in a Greek population with obesity*”, Amerikanou et al. analyzed the plasma profiles of 16 environmental metals and metalloids using a high-throughput and sensitive inductively coupled plasma mass spectrometry (ICP-MS) based approach in a Greek cohort comprising 76 individuals with obesity and metabolic disorders. After adjusting for numerous potential confounders such as gender, age, BMI, physical activity, smoking status, presence of metabolic abnormalities, and dietary intake of the corresponding metal, vanadium (V) and rubidium (Rb) were found to be negatively associated with oxidized low-density lipoprotein (Ox-LDL) levels, zinc (Zn) was negatively associated with leptin, cobalt (Co) was negatively associated with adiponectin, and both Rb and Co were positively associated with circulatory levels of neutrophil inflammatory marker myeloperoxidase (MPO). These findings underscore the dynamic relationship between environmental metals and metalloids, as well as and markers of inflammation and oxidative stress, implying a novel pathophysiological mechanism that could drive metabolic impairment.

Lastly, we thank all the contributors for enriching this Research Topic with their valuable and highly interesting publications.

## Author contributions

AK: Conceptualization, Writing – original draft, Writing – review & editing. RA: Conceptualization, Writing – review & editing. DH: Conceptualization, Writing – review & editing. FB: Conceptualization, Writing – review & editing. SS: Conceptualization, Project administration, Writing – original draft, Writing – review & editing.
